# Functional validation of the *Plasmodium falciparum* K13 C580Y mutation in recently collected Ethiopian isolates

**DOI:** 10.64898/2026.03.17.712112

**Published:** 2026-03-17

**Authors:** Angana Mukherjee, Ashenafi Bahita Assefa, Christopher V Turlo, Lisa Checkley Needham, Douglas Shoue, Tarrick Qahash, Mahlet Belachew, Dagimawie Tadesse, Enirsie Kassie, Mulu Berihun, Bokretsion G. Brhane, Jonathan B. Parr, Michael T. Ferdig, members of the MAREE Consortium, Sisay Adane, Adisu Tesfaye, Jenna Zuromski, Abebe A. Fola, Geremew Tasew, Getachew Tollera, Jonathan J. Juliano, Jeffrey A. Bailey

**Affiliations:** 1 Department of Biological Sciences, University of Notre Dame, Notre Dame, IN, USA; 2 Division of Infectious Diseases, Department of Medicine, School of Medicine, University of North Carolina at Chapel Hill, Chapel Hill, NC, USA; 3 Ethiopian Public Health Institute, Addis Ababa, Ethiopia; 4 College of Medicine and Health Sciences, Arba Minch University, Arba Minch, Ethiopia; 5 Department of Pathology and Laboratory Medicine, Brown University, Providence, RI, USA

## Abstract

Recent genomic surveillance in Ethiopia identified the first detection of the *Plasmodium falciparum* kelch13 (K13) C580Y substitution in the Horn of Africa. To assess its functional impact, we introduced C580Y into two recently collected Ethiopian clinical isolates using CRISPR-Cas9 genome editing. Ring-stage survival assays demonstrated significantly elevated *in vitro* dihydroartemisinin (DHA) survival in edited parasites relative to isogenic controls, establishing that C580Y confers artemisinin tolerance in contemporary Ethiopian genetic backgrounds, providing one of the first causal assessments of C580Y in recent African parasite isolates.

## Text

Recent genomic surveillance in northwestern Ethiopia reported the first detection of the *Plasmodium falciparum* Kelch13 (K13) C580Y substitution in the Horn of Africa, a validated molecular marker of artemisinin partial resistance (ART-R). Following its emergence in western Cambodia in the late 2000s, K13 C580Y rapidly spread (1, 2) across the Greater Mekong Subregion (GMS), reaching near-fixation in several countries and displacing other resistance-associated variants. Its repeated association with robust ART-R and efficient spread in low-transmission settings under dihydroartemisinin (DHA)–piperaquine selection highlights its evolutionary success (3, 4). For a K13 substitution to become prevalent in a parasite population, it must satisfy two minimal criteria: it must confer sufficient ART-R to provide a selective advantage under drug pressure, and it must remain compatible with asexual growth and transmission fitness. The evolutionary success of K13 C580Y in Southeast Asia may reflect this balance.

Despite the widespread emergence of multiple K13 substitutions in Africa (5–12), C580Y has remained absent. However, Zeleke *et al*. recently identified two C580Y mutant infections from Gondar Zuria district in northwestern Ethiopia, collected between November 2022 and October 2023, and confirmed by molecular inversion probe sequencing and long-read whole-genome sequencing. Haplotype analysis indicated a distinct genetic background relative to Southeast Asian C580Y lineages, marking the first detection of this mutation in the Horn of Africa to our knowledge (13).

This rare detection raises an immediate biological question: whether C580Y can resist artemether-lumefantrine pressure in patients, establish and persist in African parasite populations or, instead, its presence was transient. Addressing this question requires functional evaluation in relevant parasite genetic backgrounds, as the ART-R phenotype associated with K13 substitutions is strongly context dependent. For example,CRISPR-Cas9 introduction of prevalent Ugandan K13 substitutions A675V and C469Y into both Southeast Asian (Dd2) and East African (MAS-136) parasite lines showed that these substitutions did not uniformly confer reduced DHA susceptibility, particularly in the African background (14). These findings indicate that such K13 substitutions alone may be insufficient to generate resistance, and that permissive genetic backgrounds or additional genetic modifiers are required for phenotypic changes. Introduction of K13 C580Y has shown variable phenotype in long-adapted isolates, conferring resistance in Cambodian, Dd2 (15), Vietnamese (V1/S) (16), Ugandan isolates, and 3D7 (17), but failing to do so in others, such as Tanzanian (F32) (17) and Chinese (FCC1/HN) (18) isolates. Collectively, these studies demonstrate that the ART-R phenotypic consequences of K13 substitutions cannot be inferred from their presence alone; they must be evaluated in the context of relevant genetic backgrounds from the same region or country.

We investigated the impact of K13 C580Y by introducing it into two recently collected Ethiopian *P. falciparum* clinical isolates and assessing ART-R *in vitro*. The isolates, AM001 (Arba Minch, South Ethiopia Regional State and SK2 (Selekleka, Tigray, northern Ethiopia), were collected in 2024. Venous blood (5-7 mL) from *P. falciparum* infected patients were collected in sterile heparinized tubes, washed with incomplete RPMI medium, and cryopreserved in glycerolyte within 24 hours of collection as per WWARN guidelines. The isolates were subsequently culture-adapted *in vitro* and confirmed to carry wild-type K13 propeller sequences by Sanger sequencing (Supplementary Figure 1). Using CRISPR-Cas9 genome editing, we edited *k13* for C580Y into both these isolates. As editing controls, we also introduced two synonymous shield mutations in *k13* that do not alter amino acid sequences (19). Editing of *k13* was performed using the pDC2-coSpCas9-k13guide-gRNA-h*dhfr* all-in-one plasmid that contains a *P. falciparum* codon-optimized Cas9 sequence, a human dihydrofolate reductase (h*dhfr*) gene expression cassette (conferring resistance to WR99210) and donor template (containing the respective nonsynonymous and shield/binding site mutations or only shield/binding site mutations) (20). 80 μL of packed RBCs containing 5% ring parasitemia were electroporated with 50 μg of the purified plasmid resuspended in Cytomix using a BioRad Gene Pulser Xcell Electroporation System. Transfected parasites were maintained under 2.5 nM WR99210 (Jacobus Pharmaceuticals) to select for edited parasites until 6 days post-transfection, after which the drug was removed from the media. Parasite cultures were monitored for recrudescence microscopically for up to six weeks post electroporation.

**Figure 1:**
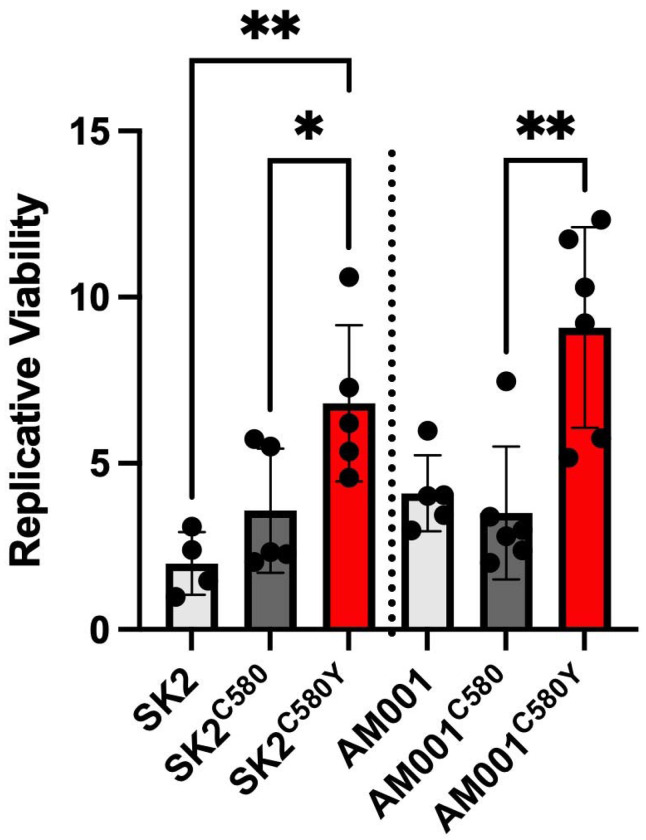
Parasite Survival after DHA exposure in CRISPR-Cas9 C580Y *k13* edited Ethiopian isolates. Mean Replicative Viability from a qPCR read out 120h post exposure to 700 nM DHA among edited parasites in 4-6 biological replicates is plotted. Error bars indicate the standard error of mean. RBCs infected with Percoll synchronized mature schizonts were incubated with uninfected RBCs for 4-6 hours, after which parasitemia was assessed by flow cytometry. 0.2% parasitemia in 2% hematocrit was exposed to 700 nM DHA or dimethylsulfoxide (DMSO, vehicle control) for 6 hours. The drug was removed by washing, and cultures were maintained for 120 hours. The numbers of intraerythrocytic parasites were measured by SYBR green in a qPCR format and replicative viability after drug treatment was calculated relative to DMSO-treated parasites. Unpaired t-tests with Welch’s correction were used to compare edited *k13* mutant strains with silent edited isogenic strains and their corresponding parental strains (SK2, a patient *P. falciparum* isolate collected from Selekleka Health Center in Tigray and AM001 from Arba Minch in South Ethiopia Regional State).

Successful edits were confirmed by Sanger sequencing (Supplementary Figure 1). To our knowledge, this represents one of the first functional validations of a K13 substitution performed through genome editing in recently collected African patient isolates. By assessing native Ethiopian parasite genetic backgrounds, this approach directly tests whether C580Y confers ART-R in epidemiologically relevant lineages and provides critical functional context for interpreting the emergence of this mutation reported by Zeleke *et al.* A DHA survival screen performed at 700 nM DHA with a qPCR readout at 120 hours post-exposure (21) demonstrated significantly elevated survival in C580Y-edited lines relative to their isogenic controls (SK2: *p* = 0.0445; AM001: *p* = 0.0031, unpaired Welch’s *t*-test), establishing that C580Y is sufficient to confer artemisinin tolerance in contemporary Ethiopian parasite backgrounds (Figure 1).

Together, these findings raise concern that C580Y in Ethiopia could contribute to clinically relevant ART-R, as previously observed in Southeast Asia. Moving forward, determining the asexual growth and transmissible fitness of these isogenic C580Y-edited lines will be essential to assess whether this newly emergent mutation is likely to persist locally or expand. Such studies will be critical for distinguishing transient emergence from evolutionary success and for anticipating the public-health significance of C580Y in Africa.

## Supplementary Material

Supplement 1
