# Supplementary material for: Functional validation of the Plasmodium falciparum K13 C580Y mutation in recently collected Ethiopian isolates
Source: bioRxiv. 2026 Mar 17:2026.03.17.712112. Preprint. [Version 1] doi: 10.64898/2026.03.17.712112 (PMC13015332; doi:10.64898/2026.03.17.712112)
Supplement: Supplement 1 [file media-1.docx]

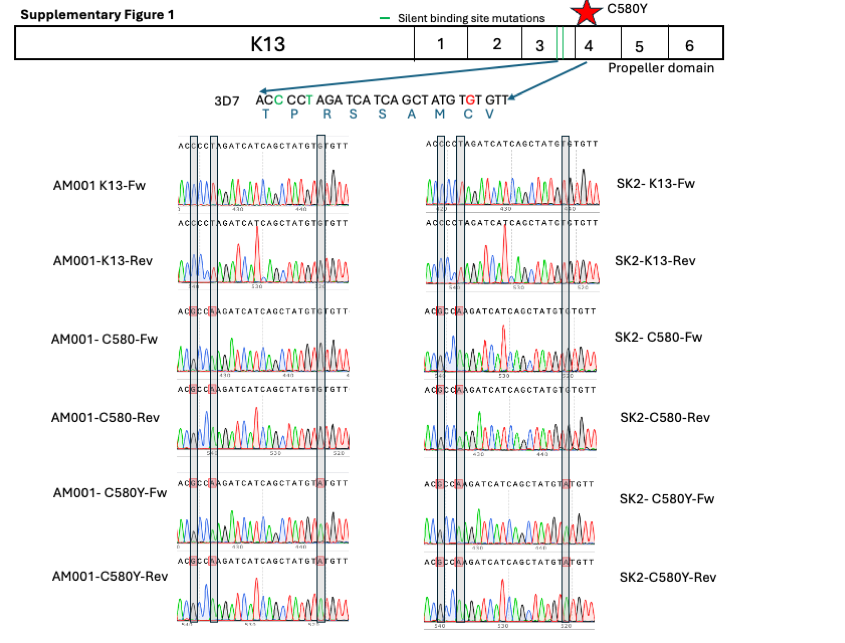


**Supplementary Figure 1:** **CRISPR-Cas9 genetic editing of K13 C580Y and silent binding site control mutations in AM001 and SK2 parasites**. Top schematic shows the locations of the C580Y and control substitutions in the K13-propeller domain. Reference DNA sequence of 3D7 is shown on top, and the Sanger sequence and chromatogram analysis of forward and reverse alleles of parent and edited lines below. Highlighted bases show the WT and edited alleles.
